# Clinicopathological and Prognostic Significance of WW Domain Binding Protein 5 Expression in Papillary Thyroid Carcinoma

**DOI:** 10.1155/2019/1791065

**Published:** 2019-11-19

**Authors:** Yangyang Qian, Zongfu Pan, Zhenying Guo, Xinyang Ge, Guowan Zheng, Jun Cao, Ping Huang, Xin Zhu, Xuhang Zhu, Qingliang Wen, Minghua Ge

**Affiliations:** ^1^The First Affiliated Hospital of Wenzhou Medical University, Wenzhou, Zhejiang 325000, China; ^2^Department of Head and Neck and Thyroid Surgery, Zhejiang Provincial People's Hospital, Hangzhou, Zhejiang 310014, China; ^3^Department of Pharmacy, Zhejiang Cancer Hospital, Hangzhou, Zhejiang 310022, China; ^4^Department of Pathology, Zhejiang Cancer Hospital, Hangzhou, Zhejiang 310022, China; ^5^Heartland Christian School, 10691 W Western Reserve Rd., Canfield, Ohio, USA; ^6^Department of Head and Neck Surgery, Zhejiang Cancer Hospital, Hangzhou, Zhejiang 310022, China

## Abstract

**Objectives:**

Many patients with papillary thyroid cancer (PTC) have a high recurrence risk and poor prognosis, and the main obstacle to the clinical diagnosis and treatment of PTC is lack of effective predictive molecular markers. The purpose of this study was to investigate the clinicopathological and prognostic implications of WW domain binding protein 5 (WBP5) expression in PTC.

**Materials and Methods:**

Immunohistochemistry of WBP5 was performed using tissue microarrays of 131 patients with PTC who underwent surgery during January 2006 and January 2010 in the Zhejiang Cancer Hospital. Statistical analyses were conducted to evaluate the association between WBP5 expression and the clinicopathological features and to analyze the disease-free survival (DFS) and prognostic factors.

**Results and Conclusion:**

The positive expression rate of WBP5 in PTC and the adjacent normal tissues was 42.75% (56/131) and 45.45% (10/22), respectively. WBP5 expression was significantly correlated with bilaterality, capsule invasion, and N-stage, and it was a favorable factor of DFS. Moreover, patients with a high WBP5 expression exhibited reduced risk of disease recurrence compared with that in patients with low WBP5 expression in the univariate analysis, whereas the multivariate analysis suggested that WBP5 was not an independent prognostic factor. Our results indicate that WBP5 might be a favorable prognosis indicator of PTC.

## 1. Introduction

Thyroid cancer (TC) is one of the most common endocrine malignancies, and its global incidence has tripled during the last three decades [1–3]. In the 2018 Global Cancer Statistics, TC was ranked the fifth most common malignancy in women, only behind breast, lung, rectal and cervical cancers [4]. Papillary thyroid carcinoma (PTC) is the most common subtype of TC, constituting approximately 80–85% of all thyroid cancer cases. Patients with PTC are typically treated by surgical resection and radioactive iodine therapy, with a five-year survival rate of over 95% [1]. In spite of the slow progression of PTC with effective treatments, approximately 15% of patients with PTC relapse within 10 years after the initial treatment, leading to aggressive disease and poor survival outcomes [5, 6]. Several clinical and molecular studies have been performed to assess the risk of PTC recurrence. The BRAF^V600E^ mutation has received great attention owing to its potential utility in identifying aggressive clinicopathological features and a high risk of recurrence in patients with PTC [7–9]. However, significant differences in the frequency of genetic alterations exist among the histologic variants of PTC [10], which might limit its clinical value in certain histologic variants. Therefore, it is important to explore novel biomarkers associated with PTC metastasis and progression.

WW domain binding protein 5 (WBP5) belongs to the WW domain binding protein family. It contains the proline-rich region and mediates the interaction of proteins [11]. WBP5 was the first of the eight ligands to be identified (WBP3 through WBP10), and it had been shown to bind to the FBP11 WW domain in a mouse limb bud expression library [12]. Studies have shown that WBP5 might induce small cell lung cancer (SCLC) multidrug resistance through the WBP5-Abl-MST2-YAP1 pathway [13–15]. In addition, WBP5 is also one of the 15 candidate oncogenes in human colorectal cancer with microsatellite instability [16]. Recently, however, WBP5 has been reported to be a possible tumor suppressor gene in gastric carcinogenesis [17]. Thus, the role of WBP5 in tumors remains controversial. In this study, we aimed to investigate the clinicopathological and prognostic implications of WW domain binding protein 5 (WBP5) expression in PTC.

## 2. Materials and Methods

### 2.1. Patients and Tissue Samples

Retrospective analysis data of patients who received primary surgical treatment for PTC between January 2006 and January 2010 were obtained. A total of 153 tissue samples were collected for this study, comprising tumor samples from 131 patients diagnosed with PTC and the adjacent normal tissue samples from 22 patients. All pathologic sections were reconfirmed by three expert pathologists. A final diagnosis was made based on postoperative histopathological examination, and some were reconfirmed by immunohistochemistry (IHC). This study had excluded patients with other types of malignancies or undergone preoperative anticancer therapy. The clinicopathological characteristics, treatment methods, and clinical outcomes were summarized according to the medical records (Table 1). The tumor-node-metastasis (TNM) stage of the patients with PTC was determined according to the eighth American Joint Committee on Cancer recommendations [18]. The surgeon decided whether or not to perform total thyroidectomy according to preoperative ultrasonography and ultrasound-guided fine needle aspiration and intraoperative exploration. All patients were treated with levothyroxine sodium tablets for thyroid hormone replacement and thyroid stimulating hormone suppression after surgery. And all the patients provied informed consent before surgical treatment, and the study was approved by the Ethics Committee of Zhejiang Cancer Hospital. The prognosis of the 131 patients with primary PTC was evaluated by regular follow-up after completion of treatment at three-month intervals in the first two years, and six-month intervals thereafter. Follow-up evaluations included clinical examination, ultrasonography, and blood tests (T3, T4, TgAb, Tg, etc.,). A chest radiograph or computed tomography (CT) was performed once yearly. Through regular review and patients with clinical suspicions of relapse were admitted to the hospital for further treatment. All cases of relapse were confirmed by pathology or imaging. We calculated the survival time interval between the diagnosis day and the latest follow-up (or relapse or death). The median duration of follow-up was 108 months (range 1–155 months). Disease-free survival (DFS) data were obtained for all the 131 (100%) patients, and 20 patients (15.3%) relapsed after surgery.

### 2.2. Tissue Microarray Construction and Immunohistochemistry

The hematoxylin and eosin-stained tissue sections were performed in the construction of tissue microarray, and 30 adjacent normal tissues and 131 PTC tissues were evaluated by three senior pathologists independently. Representative regions were selected and extracted using the TM-1 Tissue Microarray Kit (Changzhou Ruipin Precision Instrument Co. Ltd., Jiangsu, China) from each paraffin-embedded tissue block. They were subsequently embedded into blank paraffin blocks to construct the tissue microarray. The paraffin-embedded tissue microarray was incubated at 60°C for approximately 30 minutes and cooled at 25°C for better tissue fixation. A series of 3-*μ*m-thick sections were sectioned from the tissue microarray blocks and used for IHC. All paraffin-embedded sections were deparaffinized with xylene and dehydrated with alcohol. Following dehydration, microwave pre-treatment for 15 min in citrate buffer (pH 6.0) was used to retrieve the antigen. After neutralizing endogenous peroxidase with 5% hydrogen peroxide for 5 min, the sections were preincubated with WBP5 (HPA011790, ATLAS) (1 : 50 dilution) antibody at 4°C overnight. After washing with phosphate buffered saline (PBS) three times, the sections were incubated with horseradish peroxidase-labeled goat anti-rabbit secondary antibody (Dako, Glostrup, Denmark) for 50 min at 25°C, followed by three more washes in PBS. Subsequently, all the sections were visualized with 3,3′-diaminobenzidine (DAB) for 5 min, and then counterstained with hematoxylin. Immunohistochemical assessment of WBP5 expression was further evaluated by two experienced pathologists. Semiquantitative expression level of WBP5 was evaluated based on both staining intensity and percentage of positively stained cells. The staining intensity was scored as: 0 for no staining, 1for light-brown, 2 for medium-brown, and 3 for dark-brown. The percentage of staining was graded as: 0 if <5%, 1if 5% and <25%, 2 if ≥25% and <50%, 3 if ≥50% and <75%, and 4 if ≥75%. The results were classified semiquantitatively based on the multiplication of the intensity and distribution scores. Statistical analysis of the *P* values with scores of 2 (*P* = 0.0253), 3 (*P* = 0.0089), 4 (*P* = 0.0183), 5 (*P* = 0.0047) and 6 (*P* = 0.2262), indicated 5 is the best cut-off value for low expression and high expression. And a final score <5 was considered low expression and ≥5 was considered high expression.

### 2.3. RNA Extraction, Purification, Reverse Transcription, and Quantitative Real-Time Polymerase Chain Reaction (qPCR)

Human tissue sample (PTC and adjacent normal tissues) was separated and homogenized using TRIzol reagent (Invitrogen, Carlsbad, CA, USA) for the extraction of total RNA. The quality and quantity of the total RNA extracted from each sample were determined using agarose gel electrophoresis and spectrophotometry using a Nanodrop ND-1000 Spectrophotometer (Thermo Fisher Scientific Inc., USA), respectively. The mRNA was converted to cDNA using the PrimeScript RT Master Mix (TaKaRa Biotechnology, Dalian, China) according to the manufacturer's instructions. The qPCR was carried out on the LightCycler® 480 Instrument II (Roche, LightCycler 2.0, USA) using TB Green™ Premix DimerEraser™ (Code: RR091A; TaKaRa, Dalian, China). Each biological replicate was analyzed in triplicate to ensure the accuracy of the results. *β*-Actin was used as an endogenous control. The 2-^ΔΔ^CT method was used for relative quantification of gene expression.

### 2.4. Statistical Analysis

All data were analyzed using GraphPad Prism 7.04 (GraphPad Inc., La Jolla, CA, USA) and SPSS Statistics 25.0 (SPSS, Inc., Chicago, IL, USA). Univariate and multivariate analyses were performed using chi-square criterion, while the prognosis analysis was carried out using Kaplan–Meier method and Cox proportional-hazards regression models. The results with *P* values <0.05 were considered statistical significance.

### 2.5. Retrospective Analysis Using Data from TCGA

The online database Kaplan–Meier plotter (https://kmplot.com) is used to retrieve gene expression data and clinical information from the Cancer Genome Atlas (TCGA) [19]. Gene expression profiling interactive analysis (GEPIA) is a newly developed interactive web server for estimating the RNA sequencing expression data from the TCGA and Genotype-Tissue Expression (GTEx) dataset projects [20]. Candidate genes were queried by GEPIA to analyze their expression and prognostic value in clinical samples.

## 3. Results

### 3.1. WBP5 Expression Was Significantly Decreased in PTC Tissue Compared with That in the Adjacent Normal Tissues

**Figure 1:**
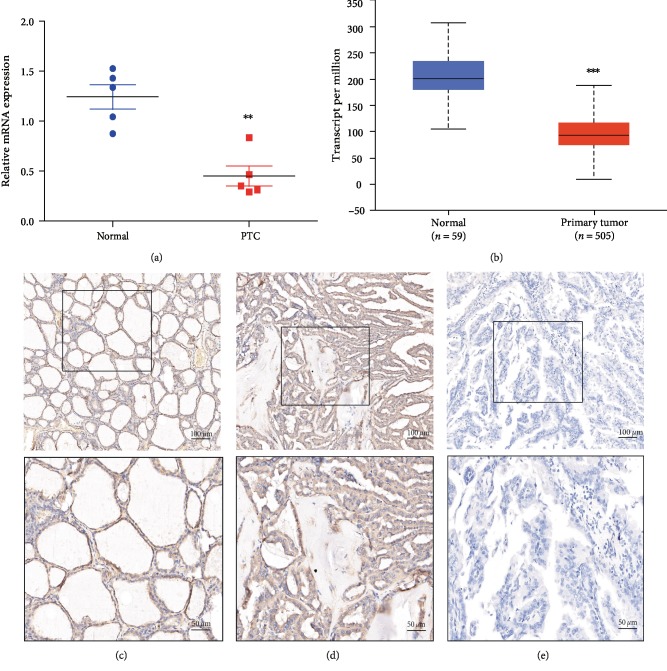
Expression of WBP5 in papillary thyroid cancer (PTC) and the paired adjacent normal tissue samples. (a) qPCR was used to detect the mRNA level of WBP5 expression in PTC and the adjacent normal tissues. ^∗∗^*P* ≤ 0.01, two-sided paired *t*-test. (b) The TCGA-thyroid carcinoma data analysis of WBP5 expression level between the normal thyroid gland versus PTC. ^∗∗∗^*P* ≤ 0.001, two-sided unpaired *t*-test. (c) Positive expression of WBP5 in the normal thyroid tissue. (d) Positive expression of WBP5 in PTC. (e) Negative expression of WBP5 in PTC.

The results of qPCR showed that the expression of WBP5 in the adjacent normal tissues was significantly higher than that in PTC (Figure 1(a)). Consistently, the TCGA-thyroid carcinoma data also suggested that the expression of WBP5 was decreased in PTC (Figure 1(b)). Moreover, WBP5 detected by IHC exhibited high expression in 45.45% of the adjacent normal tissues (10/22) and 42.75% of PTC tissues (56/131), as shown in Figures 1(c)–1(d). Finally, the immunohistochemical staining intensity of WBP5 in the adjacent normal tissues was significantly higher than that in PTC tissues.

### 3.2. Clinicopathological Implication of WBP5 Expression in PTC

The association between the expression of WBP5 and the clinicopathological features of patients with PTC is summarized in Table 1. Statistical analysis indicated that bilaterality (*P* = 0.043), capsule invasion (*P* = 0.042), and N-stage (*P* = 0.026) were significantly associated with WBP5 expression (*P* < 0.05). Notably, the postoperative recurrence rate of patients with high WBP5 expression was lower than that of patients with low WBP5 expression in PTC (*P* = 0.006). However, there was no significant correlation between WBP5 expression and age, gender, tumor number, maximal tumor diameter, and intrathyroidal dissemination.

### 3.3. Survival Analysis

**Figure 2:**
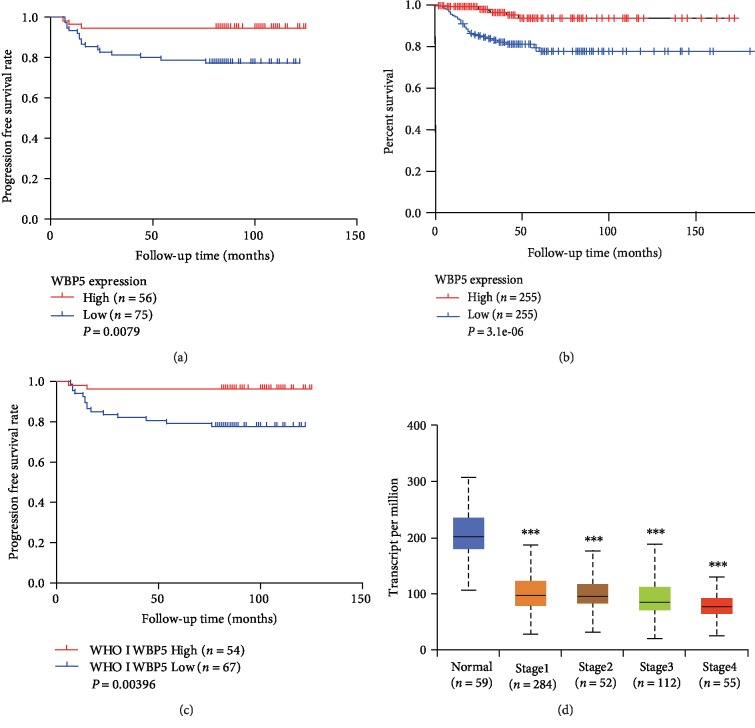
Kaplan–Meier curve and bioinformatics analyses of WBP5 expression. (a) WBP5 expression was significantly correlated with DFS in PTC (*P* < 0.05). (b) Association between WBP5 expression level and DFS in patients with PTC based on the gene expression profiling interactive analysis (GEPIA). Patients with PTC with a high WBP5 had longer DFS than those with low WBP5 expression (*P* < 0.05). (c) WBP5 expression was significantly correlated with the WHO grade I prognosis in PTC (*P* < 0.05). (d) The expression of WBP5 at different stages of PTC in the UALCAN database. ^∗∗∗^*P* ≤ 0.001, two-sided unpaired *t*-test.

As shown in Table 2, the univariate analysis revealed that patients with PTC with high WBP5 expression had lower relapse risk than those with low WBP5 expression (hazard ratio [*HR*] = 0.221, 95% confidence interval [CI]: 0.065–0.753, *P* = 0.016). Moreover, significant correlations between relapse and bilaterality (*P* = 0.003), maximal tumor diameter (*P* = 0.015), capsule invasion (*P* = 0.016), total thyroidectomy (*P* = 0.005), lymph node dissection (*P* = 0.039), and iodine radiotherapy (*P* ≤ 0.001) were also identified using univariate analysis. The subsequent multivariate analysis confirmed that both total thyroidectomy (*P* = 0.039) and iodine radiotherapy (*P* ≤ 0.001) were predictors of DFS in patients with PTC (Table 2). However, WBP5 was not identified as an independent prognostic factor using multivariate analysis (*HR* = 0.746, 95% CI: 0.191–2.921, *P* = 0.674). Kaplan–Meier analysis indicated that patients with high WBP5 expression (*P* < 0.05) had a lower risk to recur than those with low expression (Figure 2(a)). Our findings were consistent with the results of the TCGA-thyroid carcinoma data, which contained 510 samples (Figure 2(b)). Furthermore, patients with high WBP5 expression (*P* < 0.05) had a significantly longer DFS in patients with WHO grade I PTC (Figure 2(c)), while other stages were not significantly associated with WBP5 expression. In the TCGA-thyroid carcinoma data, the expression of WBP5 significantly declined in high-grade PTC compared with that in the normal thyroid tissue (Figure 2(d)).

## 4. Discussion

Currently, patients with PTC usually have a favorable prognosis. However, some patients with PTC still have a high risk of recurrence; therefore, screening for novel molecular markers of PTC recurrence is important for effective treatment and follow-up. In the present study, we found that WBP5 was highly expressed in 56 of the 131 PTC tissues (42.75%) and 10 of the 22 (45.45%) adjacent normal thyroid tissue. The data analyse showed that patients with PTC with a high WBP5 expression had a lower relapse risk than that in patients with PTC with a low WBP5 expression. Therefore, WBP5 might serve as a valuable and specific prognostic biomarker in PTC.

There have been only a few studies on WBP5, and to the best of our knowledge, the association between WBP5 and PTC clinicopathological features has not been reported. In this study, we explored the correlation between WBP5 expression and clinical features of PTC by statistical analysis of specimens from 131 patients with PTC. Analysis result suggested that WBP5 might be a favorable prognostic indicator of PTC. Interestingly, in our study, the expression of WBP5 was higher in patients with unilateral tumorigenesis, no membrane invasion, and no lymph node metastasis, suggesting that WBP5 expression is an indicator of less aggressive tumors. Nevertheless, Tang et al. [15] revealed that the expression of WBP5 might be higher in patients at the advanced disease stage than at the initial disease stage, suggesting that WBP5 can be a marker for predicting short survival time in patients with SCLC. Although it has been suggested that WBP5 might be an oncogene in human colorectal cancer with microsatellite instability [16], a recent study by Suh et al. [17] indicated that WBP5 is a possible tumor suppressor gene in gastric carcinogenesis. WBP5 (pp21 homolog) is homologous to TCEAL7 [21, 22], which is regarded as a possible tumor suppressor gene in ovarian cancer [23]. In addition, studies have reported that pp21 homolog can inhibit the activation of Rous sarcoma virus in chicken embryo fibroblasts [24]. The reason for the inconsistency in the function of WBP5 might be that WBP5 plays different roles in different tumors; there are only a few detailed studies on WBP5. In order to improve the accuracy and reliability of the usefulness of WBP5 in predicting clinical behavior of PTC, a larger sample size and extended follow-up time are needed. In the future, it is necessary to explore other functions of WBP5 in thyroid cancer, as the function of WBP5 in PTC has not been fully revealed.

## 5. Conclusions

In summary, our results suggested that WBP5 could predict favorable DFS in patients with PTC. WBP5 might serve as a valuable and specific prognostic biomarker for PTC, and WBP5 up-regulation might provide a therapeutic method for improving DFS in patients with PTC.

## Figures and Tables

**Table 1:** Association of WBP5 expression with clinicopathological factors in 131 PTC patients.

Variables	WBP5		*P*
	Low (*n* = 75)	High (*n* = 56)	
*Age (years)*			
<55	65	52	0.257
≥55	10	4	

*Gender*			
Men	18	11	0.301
Women	47	45	

*Histological variants*			
PTC			
Classical variant	63	52	0.054
Follicular variant (infiltrative)	12	3	
Solid variant	0	1	

*Bilaterality*			
Unilateral	53	48	0.043^∗^
Bilateral	22	8	

*Tumor number*			
Solitary	47	43	0.085
Multiple	28	13	

*Maximal tumor diameter*			
<1 (cm)	17	21	0.111
1~4 (cm)	51	33	
>4 (cm)	7	2	

*Caspsule invasion*			
Absent	33	37	0.042^∗^
Present	10	4	
Extracapsular	32	15	

*Intrathyroidal dissemination*			
Absent	61	50	0.211
Present	14	6	

*T staging*			
pT1	35	35	0.195
pT2	6	4	
pT3	18	12	
pT4	16	5	

*N staging*			
pN0/Nx	24	29	0.026^∗^
pN1a	29	20	
pN1b	22	7	

*M staging*			
M0	74	54	0.797
M1	1	2	

*TNM staging*			
Ⅰ	67	54	0.562
Ⅱ	6	2	
Ⅲ	1	0	
Ⅳ	1	0	

*Total thyroidectomy*			
Not done	49	46	0.033^∗^
Done	26	10	

*Lymph node dissection*			
Not done	9	4	0.261
CCND only	35	34	
CCND with MRND	31	18	

*Iodine radiotherapy*			
Not done	47	52	0^∗^
Done	28	4	

*Recurrence of disease*			
Absent	58	53	0.006^∗^
Present	17	3	

^a^PTC, papillary thyroid carcinoma, ^b^CCND, central compartment node dissection, ^c^MRND, modified radical neck dissection, ^∗^Significantly different by the χ^2^ test.

**Table 2:** Univariate and multivariate cox regression analysis of WBP5 expression with patient prognosis.

Variable	Univariate analysis		Multivariate analysis	
	HR (95% CI)	*P*-value	HR (95% CI)	*P*-value
Age (years)	0.969 (0.933–1.007)	0.106		
Gender	1.642 (0.631–4.272)	0.310		
Histological variants	0.048 (0.000–9.588)	0.262		
Bilaterality	3.834 (1.593–9.226)	0.003^∗^	2.704 (0.555–13.183)	0.218
Tumor number	1.555 (0.635–3.803)	0.334		
Maximal tumor diameter	2.735 (1.214–6.161)	0.015^∗^	1.364 (0.458–4.058)	0.577
Capsule invasion	1.826 (1.121–2.975)	0.016^∗^	1.088 (0.552–2.145)	0.808
Intrathyroidal dissemination	2.020 (0.734–5.559)	0.173		
TNM staging (I/II VS III/IV)	0.049 (0.000–234490.651)	0.700		
Total thyroidectomy	3.580 (1.482–8.650)	0.005^∗^	0.138 (0.021–0.902)	0.039^∗^
Lymph node dissection	2.297 (1.044–5.055)	0.039^∗^	1.053 (0.469–2.362)	0.900
Iodine radiotherapy	15.265 (5.086–45.813)	≤0.001^∗^	26.947 (6.232–116.515)	≤0.001^∗^
WBP5 expression	0.221 (0.065–0.753)	0.016^∗^	0.746 (0.191–2.921)	0.674

HR : Hazard ratio. ^∗^Statistical significance.

## Data Availability

The data that support the findings of this study are available from the corresponding author, Minghua Ge, upon reasonable request.
